# Supplementary figures and images for: Sirtuin 2 promotes human cytomegalovirus replication by regulating cell cycle progression
Source: mSystems. 2023 Nov 2;8(6):e00510-23. doi: 10.1128/msystems.00510-23 (PMC10734535; doi:10.1128/msystems.00510-23)

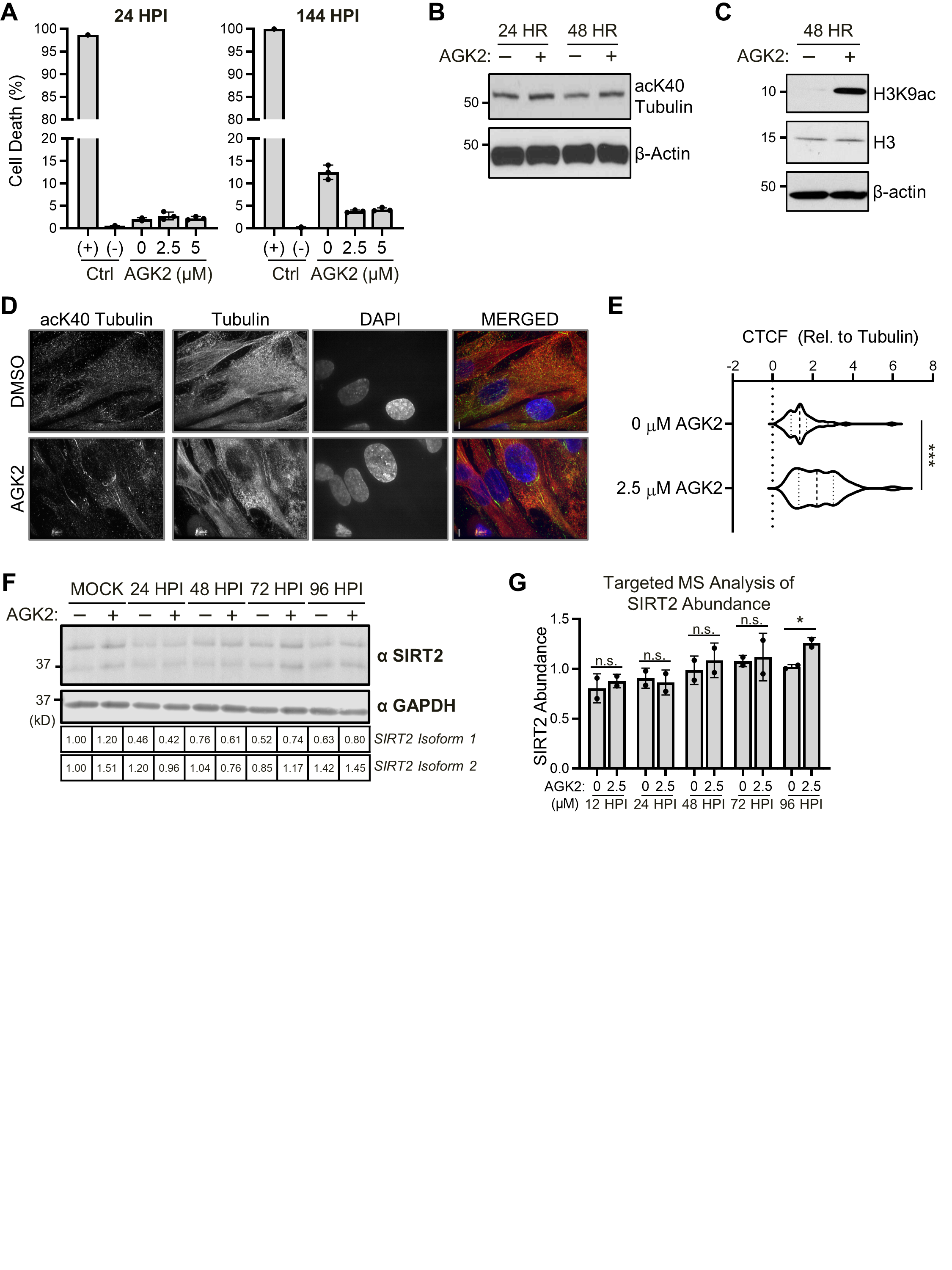

Supplement: Fig. S1 — Validation of AGK2 efficacy in human fibroblast cell culture model. [file msystems.00510-23-s0001.tif]

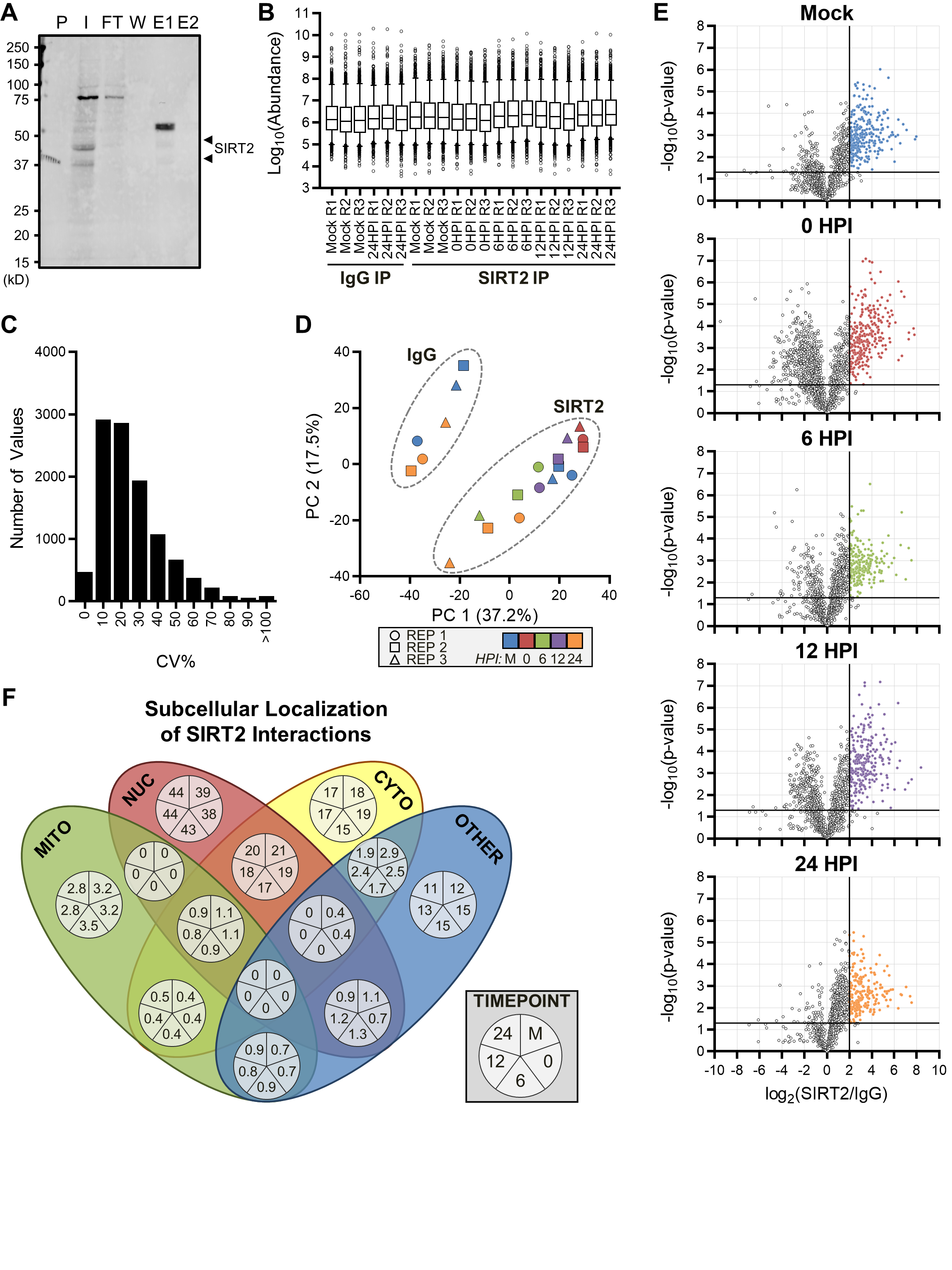

Supplement: Fig. S2 — Assessment of SIRT2 immunoaffinity purification efficiency and IP-MS data quality. [file msystems.00510-23-s0002.tif]

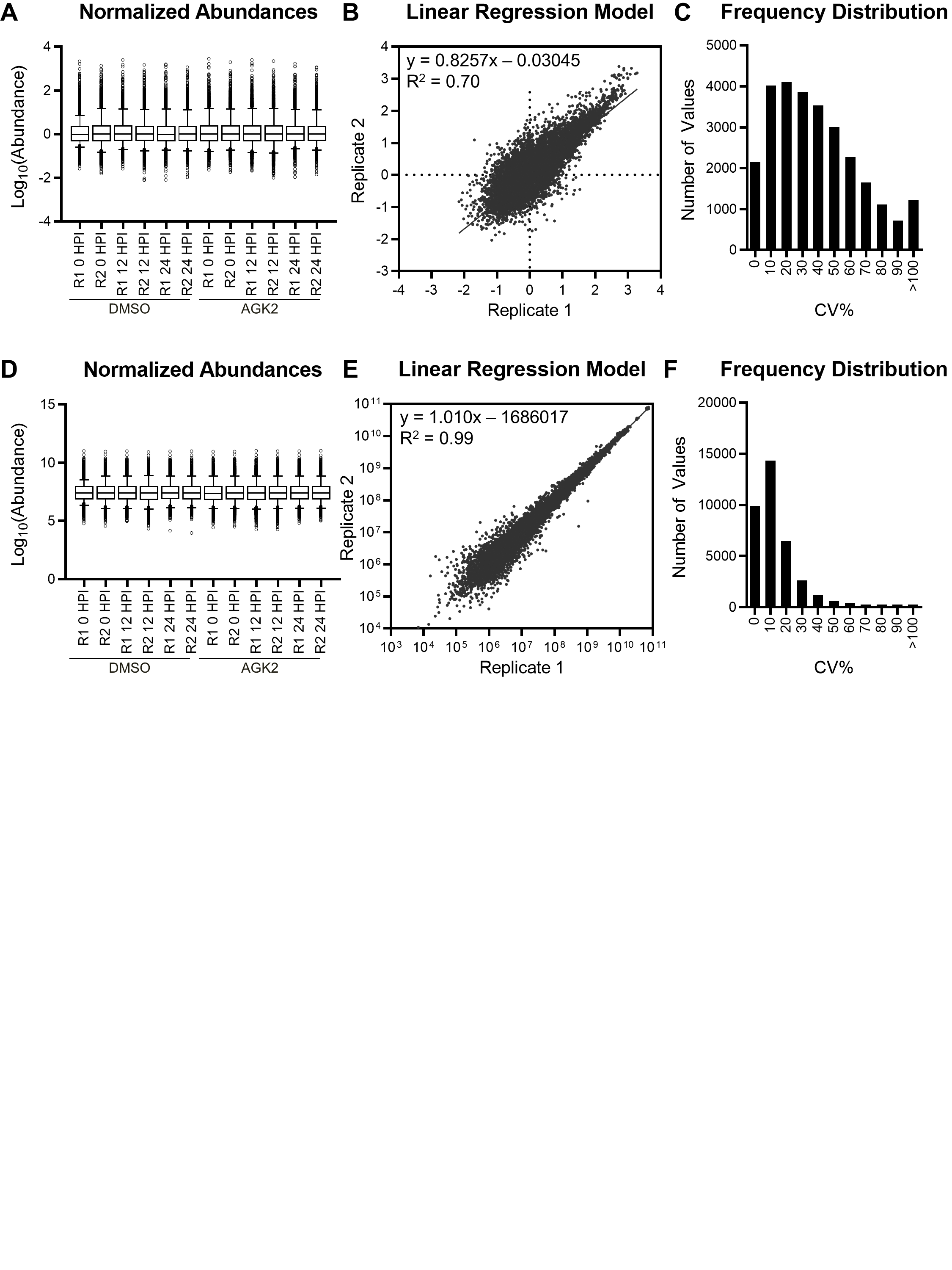

Supplement: Fig. S3 — Assessment of SIRT2 immunoaffinity purification efficiency and IP-MS data quality. [file msystems.00510-23-s0003.tif]

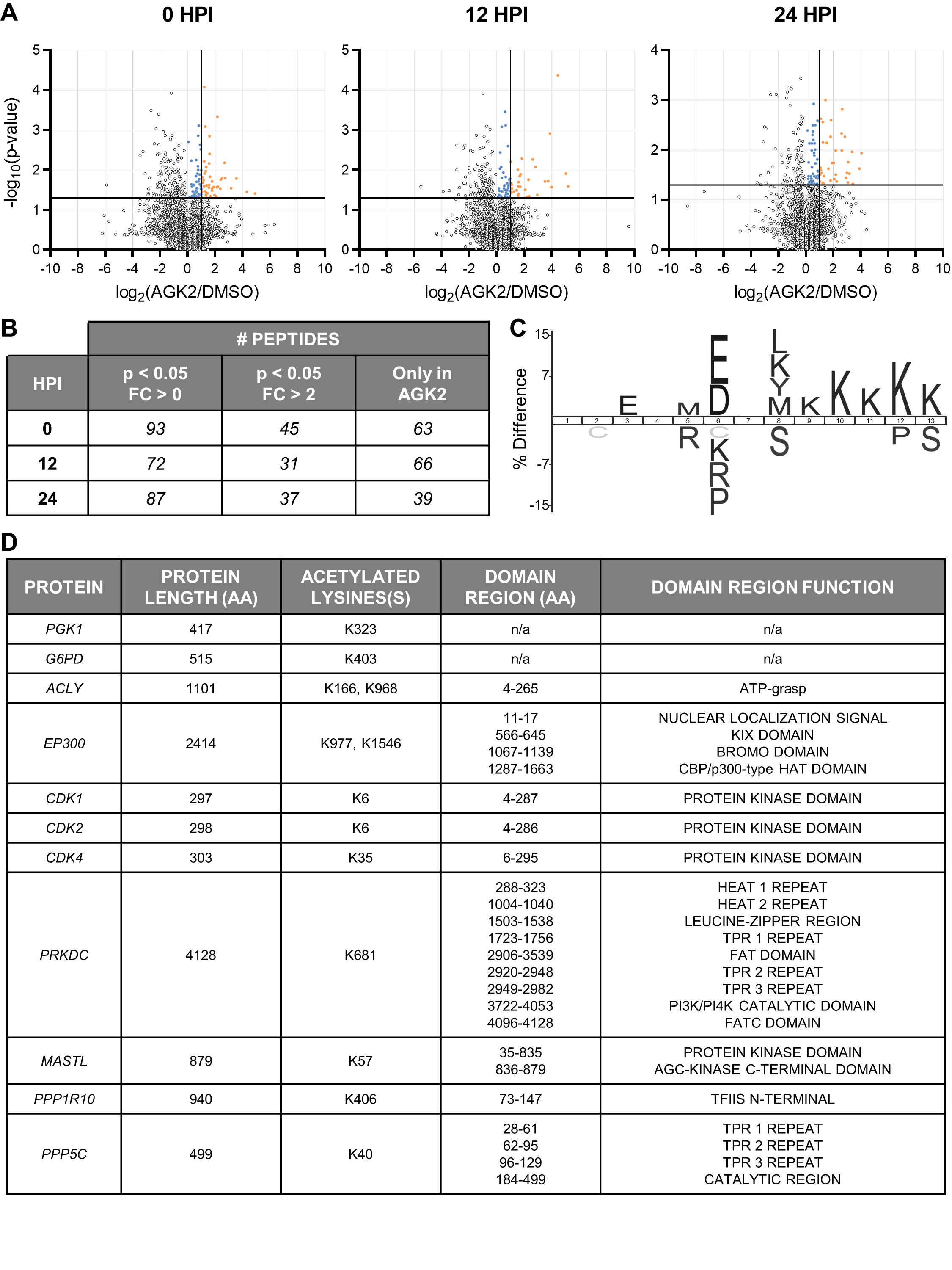

Supplement: Fig. S4 — Analysis of proteins with acetylation sites that increased in abundance following SIRT2 inhibition. [file msystems.00510-23-s0004.tif]

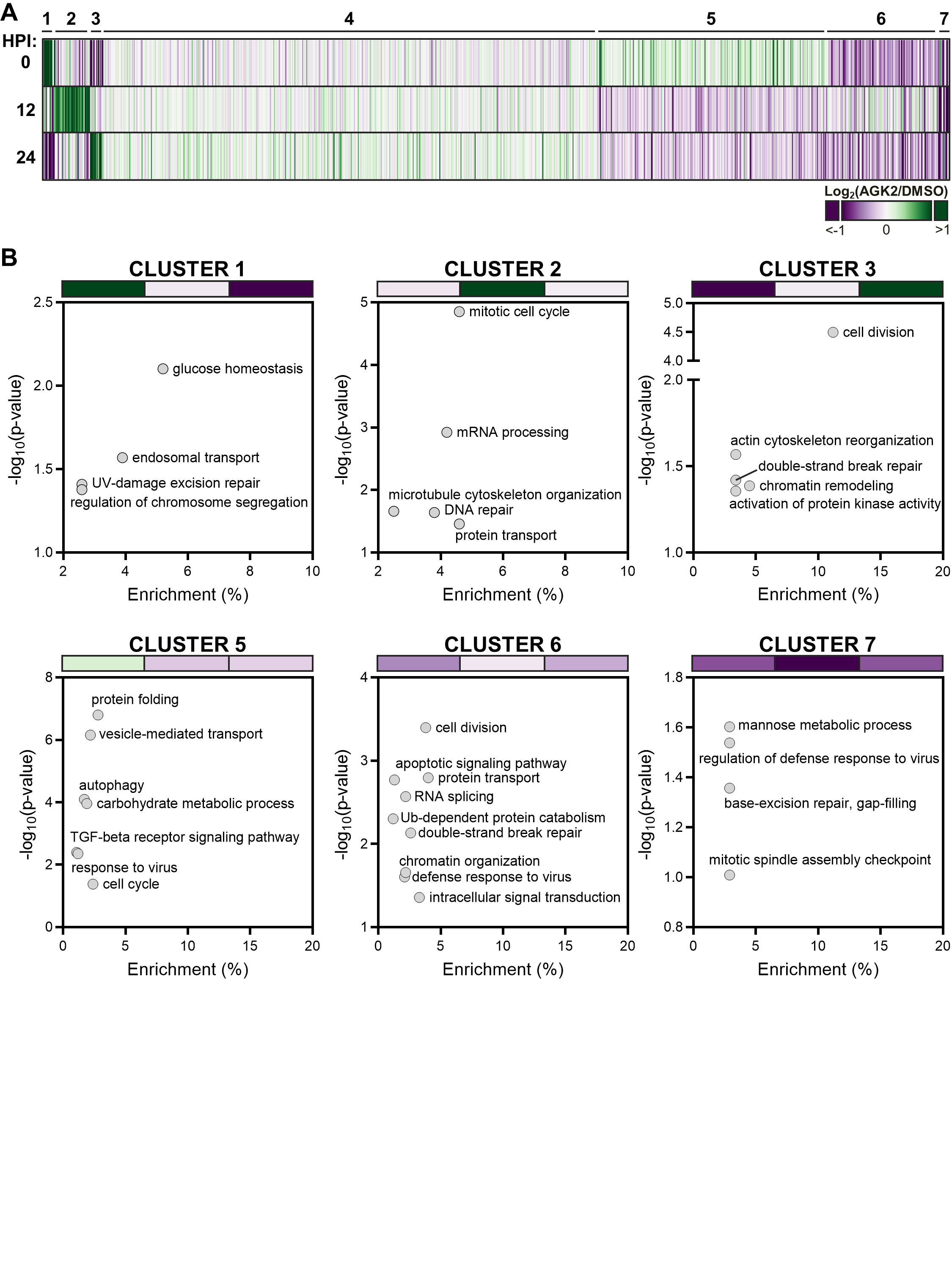

Supplement: Fig. S5 — Analysis of cellular proteome alterations driven by SIRT2 inhibition. [file msystems.00510-23-s0005.tif]

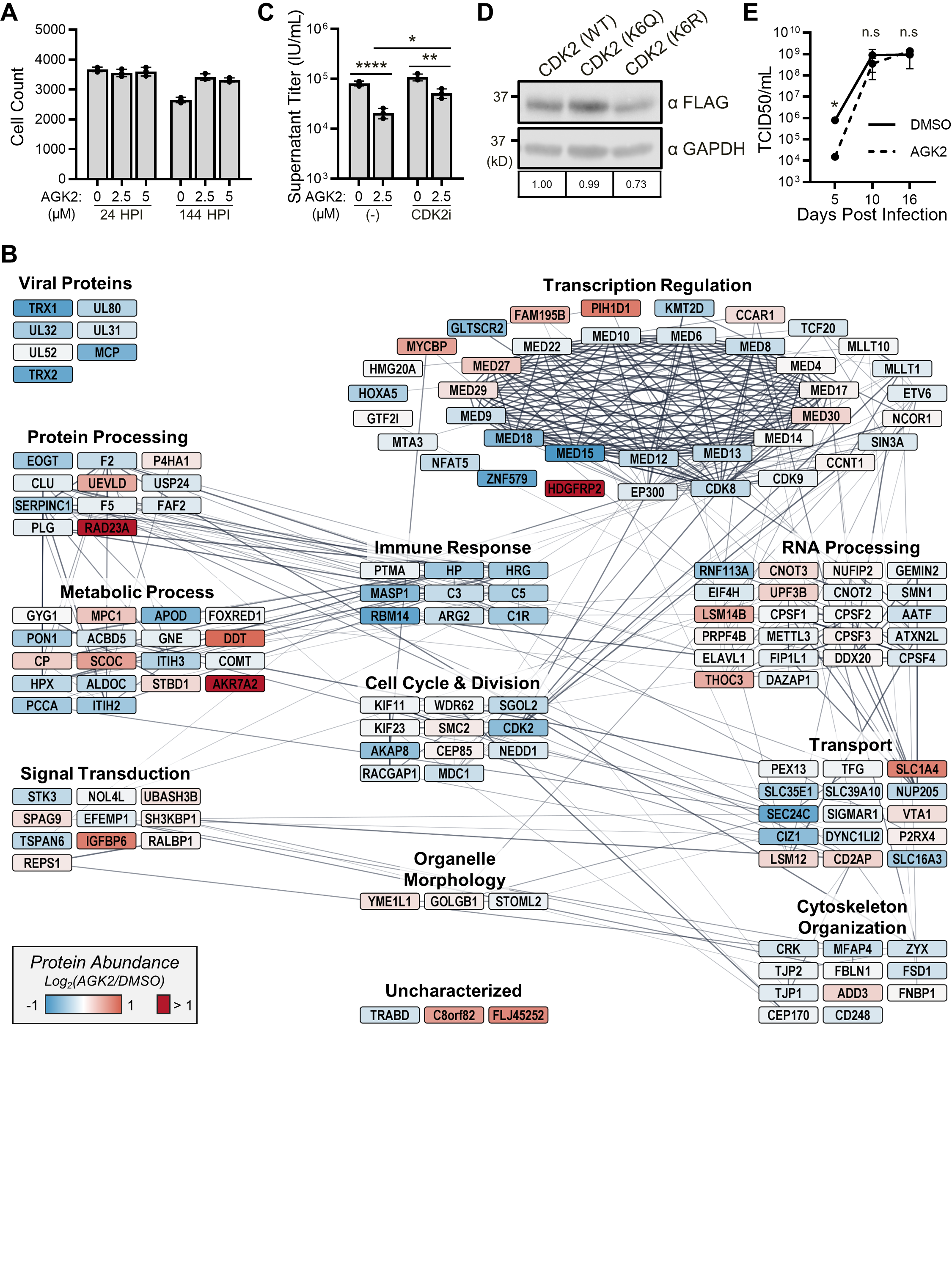

Supplement: Fig. S6 — Assessment of the impact of SIRT2-mediated cell cycle regulation on HCMV replication. [file msystems.00510-23-s0006.tif]
